# Aneurism of Femoral Artery Cured by Ligature

**Published:** 1864-03

**Authors:** E. Burke Haywood

**Affiliations:** General Hospital No. 7, Raleigh, North Carolina.


ART. III.—*Aneurism of Femoral Artery cured by Ligature.* Reported by Surgeon E. BURKE HAYWOOD, General Hospital No. 7, Raleigh, North Carolina.

Dixon Spivey, private Co. "C," 5th N. C T., was sent to this hospital, May 28th, 1863. He was a large muscular man, by occupation a farmer, age 46 years. He was shot, while a deserter, by a militia officer, and was wounded with buckshot—six shot penetrating the left thigh about the middle, and two the right leg. Traumatic aneurism of the femoral artery supervened on the 9th of June, 1863, produced by one of the wounds of the left thigh. The aneurism was seven inches above the knee. It was three inches in breadth and three and one-fourth inches in length. Pulsation very evident and strong. He having lost a great deal of blood when shot, I put him on quinia, iron, and generous diet, perfect rest of limb with cold applications. June 11th, I commenced digital compression of the femoral artery, immediately below Poupart's ligament. The compression was kept up, so as greatly to diminish, for twelve hours and a half, the current of blood. I gave, at the same time, tincture veratrum viride. At the end of twelve hours and a half, the compression was discontinued, and I had the pleasure of not being able to detect pulsation in the tumor. I think a coagulum was formed as no pulsation was perceptible up to the 3d of July, 1863, when a slight pulsation could be felt, which gradually grew stronger. The tumor also increased in size. He complained of much pain in the limb. The pulsation became stronger than ever, and the aneurismal tumor larger. The whole limb became swollen and œdematus. July 8th, 1863, I determined to ligate the femoral artery. I ligated it in Scarpa's triangle, five inches from its origin—the Sartorius muscle being drawn outwards during the operation. The operation has been, so far, successful, and the patient is convalescing. I gave him chloroform. What is most remarkable in this case is, that pulsation should have returned in the aneurismal tumor, after its suspension from the 11th of June to the 3d of July.

Jan. 10th, 1864.—I saw Spivey to-day. He has had no return of the aneurism. The operation was entirely successful.

